# Perceptual processing during trauma, priming and the development of intrusive memories

**DOI:** 10.1016/j.jbtep.2012.10.001

**Published:** 2013-06

**Authors:** Oliver Sündermann, Marit Hauschildt, Anke Ehlers

**Affiliations:** a Department of Psychology, King's College London, Institute of Psychiatry, London, UK; b Department of Experimental Psychology, University of Oxford, South Parks Road, Oxford OX1 3UD, UK

**Keywords:** Trauma memories, Enhanced perceptual priming, Posttraumatic stress disorder, Data-driven processing, Dissociation

## Abstract

**Background:**

Intrusive reexperiencing in posttraumatic stress disorder (PTSD) is commonly triggered by stimuli with perceptual similarity to those present during the trauma. Information processing theories suggest that perceptual processing during the trauma and enhanced perceptual priming contribute to the easy triggering of intrusive memories by these cues.

**Methods:**

Healthy volunteers (*N* = 51) watched neutral and trauma picture stories on a computer screen. Neutral objects that were unrelated to the content of the stories briefly appeared in the interval between the pictures. Dissociation and data-driven processing (as indicators of perceptual processing) and state anxiety during the stories were assessed with self-report questionnaires. After filler tasks, participants completed a blurred object identification task to assess priming and a recognition memory task. Intrusive memories were assessed with telephone interviews 2 weeks and 3 months later.

**Results:**

Neutral objects were more strongly primed if they occurred in the context of trauma stories than if they occurred during neutral stories, although the effect size was only moderate $\left({\eta_{p}}^{2}=.08\right)$ and only significant when trauma stories were presented first. Regardless of story order, enhanced perceptual priming predicted intrusive memories at 2-week follow-up (*N* = 51), but not at 3 months (*n* = 40). Data-driven processing, dissociation and anxiety increases during the trauma stories also predicted intrusive memories. Enhanced perceptual priming and data-driven processing were associated with lower verbal intelligence.

**Limitations:**

It is unclear to what extent these findings generalize to real-life traumatic events and whether they are specific to negative emotional events.

**Conclusions:**

The results provide some support for the role of perceptual processing and perceptual priming in reexperiencing symptoms.

## Highlights

► We investigated whether perceptual processing and priming predict intrusive memories. ► Neutral objects were more readily identified if previously seen in a traumatic rather than neutral context. ► Enhanced perceptual priming predicted intrusive memories 2 weeks later. ► Perceptual processing during the stories also predicted intrusive memories. ► The results support the role of perceptual triggers of unwanted trauma memories.

## 1 Introduction

Intrusive memories are considered the hallmark symptom of posttraumatic stress disorder (PTSD). They commonly consist of relatively brief sensory impressions from the trauma (Hackmann, Ehlers, Speckens, & Clark, 2004; Reynolds & Brewin, 1999; Van der Kolk & Fisler, 1995), that are experienced as happening in the “here and now” rather than being a memory from the past (Hackmann et al., 2004; Michael, Ehlers, Halligan, & Clark, 2005), and are easily triggered by a wide range of internal and external cues (Southwick et al., 1993; Van der Kolk & Fisler, 1995). Intrusive memories and their triggers can include those that bear no meaningful relationship with the traumatic event but are only temporally associated (Ehlers & Clark, 2000; Ehlers et al., 2002; Speckens, Ehlers, Hackmann, Ruths, & Clark, 2007). The present experiment investigated two related processes that may be involved in the easy triggering of intrusive memories, perceptual processing during the trauma and perceptual priming.

### 1.1 Perceptual priming

Priming is a type of implicit memory that is characterized by enhanced identification of previously seen stimuli (Schacter, 1992). Ehlers and Clark (2000) proposed that the easy triggering of intrusive memories by perceptually similar stimuli is, in part, a function of *strong perceptual priming*. They suggested that trauma survivors who acquire strong priming for stimuli that they encountered during the traumatic event have a reduced perceptual threshold for these stimuli. This makes it more likely that they detect potential triggers of intrusive memories in their environment.

There is preliminary evidence for a role of perceptual priming in PTSD. Several studies found that after encoding trauma-related and neutral material, people with PTSD show greater perceptual priming for trauma-related material compared to controls in word-stem completion or perceptual identification tasks (e.g., Amir, Leiner, & Bomyea, 2010; Ehring & Ehlers, 2011; Michael, Ehlers, & Halligan, 2005). Priming predicted PTSD severity 6 months later (Ehring & Ehlers, 2011; Michael, Ehlers, & Halligan, 2005). Kleim, Ehring, and Ehlers (2012) further found that trauma survivors with PTSD show perceptual processing advantages in identifying blurred trauma-related pictures compared to those without PTSD.

Experimental analogue studies provided initial support for the role of perceptual priming in the development of intrusive trauma memories (Arntz, de Groot, & Kindt, 2005; Ehlers, Mauchnik, & Handley, in press; Ehlers, Michael, Chen, Payne, & Shan, 2006; Michael & Ehlers, 2007). For example, a paradigm developed by Ehlers et al. (2006) investigates priming for neutral objects that are perceived just before something “traumatic” happens. Participants watch a series of “traumatic” and neutral picture stories, each comprising three pictures. The content of the first picture is neutral, introduces the main character of the story, and contains neutral objects for which priming was later measured. The plot then unfolds in the second picture either in a traumatic or in a neutral way. The last picture depicts the outcome of the story. With this paradigm, neutral stimuli that preceded a “traumatic” event showed enhanced perceptual priming and predicted intrusive memories (Ehlers et al., 2006; Michael & Ehlers, 2007).

### 1.2 Perceptual processing during trauma

Information processing models of PTSD suggest that strong encoding of perceptual information and relatively weak encoding of contextual information during trauma predicts subsequent reexperiencing symptoms (Brewin, Dalgleish, & Joseph, 1996; Brewin, Gregory, Lipton, & Burgess, 2010; Ehlers & Clark, 2000). Two related ways of processing that may facilitate strong encoding of perceptual information have been investigated in PTSD; (i) data-driven processing (Roediger, 1990) refers to encoding that focuses on the surface level features of a situation (i.e., sensory details such as colours and shapes) rather than its meaning (Ehlers & Clark, 2000), and (ii) dissociation is a complex concept involving a lack of integration of subjective experiences including depersonalization, derealization, altered time perception and emotional numbing (e.g., Van der Kolk & Fisler, 1995). It has been suggested that dissociation during trauma may in part predict PTSD because, like data-driven processing, it leads to preferential encoding of perceptual information (Ehlers & Clark, 2000). It has further been suggested that dissociation during a traumatic event decreases focal attention, thereby interfering with meaningful processing of the traumatic event and promoting a nonverbal, perceptual processing style (Brewin et al., 1996; Siegel, 1995).

Clinical and analogue studies found that data-driven processing and dissociation predict later reexperiencing and PTSD (e.g., Ehring, Ehlers, & Glucksman, 2008; Halligan, Michael, Clark, & Ehlers, 2003; Holmes, Brewin, & Hennessy, 2004). It is not yet clear by which mechanisms these forms of processing during the trauma contribute to PTSD. One possible pathway is perceptual priming (Ehlers & Clark, 2000) which is thought to rely heavily on perceptual operations and unconscious processes and should therefore benefit from encoding styles that favour perceptual information (Roediger, 1990). In line with this hypothesis, data-driven processing and dissociation correlated with priming in clinical and analogue studies (Ehlers et al., 2006; Lyttle, Dorahy, Hanna, & Huntjens, 2010; Michael & Ehlers, 2007).

### 1.3 Aims of the study

A new version of Ehlers et al.'s (2006) paradigm to investigate perceptual priming for objects from traumatic contexts and its relationship with processing styles and intrusions was developed to address some limitations of the earlier paradigm. First, the number of primed neutral objects was increased. Second, objects were no longer presented *within* the slides of the picture stories, but interspersed in the intervals between story pictures so that they could be counterbalanced across trauma and neutral stories.

The study investigated the following hypotheses:

Hypothesis 1 Neutral stimuli are more strongly primed if they occur in a traumatic context than if they occur in a neutral context.

Hypothesis 2 Enhanced perceptual priming for objects previously seen in a traumatic context predicts subsequent intrusive memories.

Hypothesis 3 Data-driven processing and state dissociation during traumatic stories predict subsequent intrusive memories.

Hypothesis 4 Data-driven processing and state dissociation predict enhanced perceptual priming for trauma-related stimuli.

In addition, we assessed recognition memory for objects previously seen in traumatic and neutral contexts to check the possible influence of explicit memory on priming effects. On the basis of previous results with a similar paradigm (Ehlers et al., 2006; Michael & Ehlers, 2007), we did not expect an influence of story context on recognition memory. We also assessed state anxiety and verbal intelligence to explore their relationship with priming and intrusive memories.

## 2 Material and methods

### 2.1 Participants

Fifty-six healthy volunteers were recruited from a database of people interested in participating in research. Potential participants were excluded if they had a history of past trauma (*n* = 0), were currently suffering from depression or blood/injury phobia (*n* = 0), had previous experience with related psychological experiments (*n* = 3) and/or had frequent previous exposure to distressing visual stimuli (e.g., professional background involving exposure to dead bodies or photos of mutilated bodies, *n* = 2). Thus, the final sample comprised 51 participants (27 male). Ages ranged from 18 to 68 years (*M* = 30.6; *SD* = 11.4). All participants completed the first follow-up interview at 2 weeks after the experiment (100%); and 41 (80%) participated in the second follow-up interview after 3 months. Participants received £15 as reimbursement for their time and travel expenses.

### 2.2 Experimental paradigms

The experiment was programmed with SuperLab 4.0 (Cedrus Corp, San Pedro, CA, USA). Picture stories and memory tests were presented on a 17″ Dell monitor using a Dell Optiplex PC (GX 270).

#### 2.2.1 Picture stories

Participants watched two trauma and two neutral picture stories comprising eight pictures on a computer screen, and listened to a narrative of what was happening in the story. Presentation mode was adapted from Cahill, Prins, Weber, and McGaugh (1994) and Heuer and Reisberg (1990). Each picture was presented for 15 s, and the narrative started 3 s into the picture. Table 1 shows the structure of the stories. A total of four different trauma stories and parallel neutral stories were constructed for the experiment. The parallel neutral stories started with the same 3 pictures as the respective trauma story to introduce the main characters, and then unfolded in a neutral way over the remaining 5 pictures.

Participants were informed that the purpose of the experiment was to study emotional effects of picture stories and were instructed to watch the pictures closely and imagine that they were an observer present at the scene. Neutral and trauma stories were matched in terms of number of people in the stories and gender of characters.

Each participant saw two trauma stories and the two neutral stories that were parallel to the remaining trauma stories. To minimize mood carry-over effects between stories, trauma and neutral stories were presented blockwise and separated by a 10-min break during which participants completed questionnaires. Trauma and neutral story blocks were counter-balanced across participants. Furthermore, within blocks, the order of the two picture stories was counter-balanced.

#### 2.2.2 Interspersed objects

In the interval between the pictures of the picture stories, after 1 s of a blank screen, photos of neutral objects (e.g., spirit level, onion) that were unrelated to the content of the story briefly appeared on the computer screen for 2 s. These neutral objects were of main interest to the experiment because priming and recognition memory for these objects was later measured. Participants were instructed that some other pictures would occur in between the snapshots of the story, that these were unrelated to the story and that they did not need to take any notice of them but instead should focus on the story.

There were two parallel sets of 14 neutral objects that were selected in pilot studies (see Section 2.2.4). Object sets were counter-balanced across the trauma and neutral stories. Furthermore, the position of objects *within the stories* was counter-balanced with a Latin Square design so that across participants each object occurred in each position during the stories.

#### 2.2.3 Memory tests

Two forms of memory for the interspersed objects were assessed, perceptual priming and recognition memory.

##### 2.2.3.1 Perceptual priming task

Perceptual priming was assessed with a blurred object identification task. Blurred pictures of the 28 objects previously shown in the picture stories and 28 new (unprimed) objects were presented in random order. The participants' task was to identify and name the objects as quickly and as precisely as possible. Participants were not informed that the test was an implicit memory test. They were told that this was a new and unrelated task that aimed to assess how easily blurred pictures can be identified. Answers were tape-recorded and written down and later scored for accuracy. The dependent measure was the rate of correct identification. For correlational analyses, an enhanced perceptual priming score was computed as EPP = identification rate (objects previously seen in traumatic context) minus identification rate (objects previously seen in neutral context), signifying the enhanced priming linked to traumatic contexts.

##### 2.2.3.2 Object recognition task

Recognition memory for the interspersed objects was assessed with an object recognition task. This task was incorporated into the study to assess the possible influence of explicit memory processes on the priming measure and intrusive memories. Participants were shown the original (unblurred) objects that had been interspersed into the picture stories and new control objects for 2 s in random order. The participants' task was to indicate for each object whether or not they had seen it within the picture stories.

Scoring of the object recognition test followed signal detection theory (Macmillan & Creelman, 1991). From the hits (original objects correctly identified as “old”) and false alarms (objects erroneously identified as “old”), sensitivity (d′) and response bias (*c*) scores were calculated. Sensitivity indicates how well participants discriminated between objects from the picture stories and “new” parallel objects, d′ = probit (hits) − probit (false alarms). Response bias. a measure of leniency in endorsing an object as “old”, is calculated as *c* = −.5* (probit (hits) + probit (false alarms)).

#### 2.2.4 Pilot studies of stimulus material

Three pilot studies were conducted to assess the suitability of the newly developed stimulus material.

##### 2.2.4.1 Pilot study 1: baseline identification rates of blurred objects

The aim of the first pilot study (*N* = 34*)* was to adjust baseline identification rates of the blurred objects to a degree which allowed approximately 40–50% correct identification of the objects *without prior encoding*. On the basis of the responses of the first 14 participants, blurredness was systematically adjusted with a Gaussian filter (Adobe Photoshop, Version 9.0) using a radius between 8 and 15 pixels. All objects were sized between 190 and 300 × 181–240 pixels. The remaining 20 participants provided the baseline identification rates for the two picture sets of objects (A and B) were as follows. Set A: *M* = .41, *SD* = .10; Set B: *M* = .41, *SD* = .10; *t*(19) = .33, *p* = .75, ${\eta_{p}}^{2}=.00$.

##### 2.2.4.2 Pilot study 2: identification rates when encoded without emotional context

To ensure that objects from both picture sets were not only similar in baseline identification rates, but also in salience, a second pilot study (*N* = 20*)* measured identification rates of the two sets of blurred objects following prior encoding without any (emotional) context. The unblurred objects were presented in random successive order. After a distraction phase (unrelated visual filler tasks), the neutral objects were presented again, this time blurred, and participants had to identify them as quickly and precisely as possible. There were no differences in identification rates between the sets: Set A: *M* = .60, *SD* = .11; Set B: *M* = .60, *SD* = .11; *t*(19) = −.81, *p* = .429, ${\eta_{p}}^{2}=.00$. Hence, possible memory differences for the interspersed objects reported in this study can be attributed to the emotional nature of the picture stories.

##### 2.2.4.3 Pilot study 3: suitability of picture stories

Participants (*N* = 27) rated each story on scales from −10 to +10 in terms of pleasantness (*extremely unpleasant* to *very pleasant*) and activation (*very relaxing* to *very activating*). Traumatic stories were perceived as more activating (*M* = 5.0, *SD* = 3.1 vs. *M* = −7.9, *SD* = 1.4; *F* (1,26) = 68.4, *p* < .001, ${\eta_{p}}^{2}=.74$) and more unpleasant (*M* = −3.0, *SD* = 2.8 vs. *M* = 4.9, *SD* = 2.6; *F* (1, 26) = 303.5, *p* < .001, ${\eta_{p}}^{2}=.93$) than neutral stories. No differences between trauma and neutral stories were found in the participants' reported ability to keep their mind focused on the stories (*M* = 8.8, *SD* = 1.0 vs. *M* = 8.4, *SD* = 1.5; *F*(1, 26) = 3.5, *p* = .08, ${\eta_{p}}^{2}=.13$) and to imagine being an observer at the scene of the story (*M* = 7.3, *SD* = 1.9 vs. *M* = 7.2, *SD* = 2.2; *F*(1, 26) = .2, *p* = .68, ${\eta_{p}}^{2}=.01$), each on scales from 0 *not at all* to 10 *very much*. Furthermore, participants engaged significantly more in data-driven processing during traumatic picture stories than in neutral stories on a scale from 0 to 4 (*M* = 0.9, *SD* = 0.5 vs. *M* = 0.7, *SD* = 0.4; *F*(1, 26) = 8.8, *p* = .007, ${\eta_{p}}^{2}=.28$).

### 2.3 Self-report measures

#### 2.3.1 Manipulation checks

After each picture story, participants rated the story on scales from −10 to +10 in terms of pleasantness (*extremely unpleasant* to *very pleasant*) and activation (*very relaxing* to *very activating,* e.g.*, pounding heart, tense muscles*).

After the priming task, a short interview assessed whether participants noticed that some blurred objects had earlier occurred in the picture stories, and if so, whether they tried to *actively recall* the objects during the blurred picture task.

#### 2.3.2 Data-driven processing and dissociation questionnaires

The 8-item *Data-driven Processing Scale* (DDPS, Halligan, Clark, & Ehlers, 2002) assessed processing of perceptual features of the picture stories (e.g., “*It was like a stream of unconnected impressions following each other*”). The 9-item *State Dissociation Questionnaire* (SDQ, Murray, Ehlers, & Mayou, 2002) assessed different aspects of dissociation (e.g., *“I felt distant from my emotions”*). Items on both scales were rated from 0 *not at all* to 4 *very much*, and the total score was the mean of all items. Previous studies showed satisfactory to good psychometric properties for both scales (internal consistencies above .70; convergent validity with related measures above .50), and discriminated between participants with and without PTSD symptoms in traumatized and non-traumatized samples (e.g., Ehring et al., 2008; Halligan et al., 2002; 2003; Murray et al., 2002). Internal consistencies of the DDPS and the SDQ in the present sample were *α* = .71 and *α* = .73 for trauma stories, and *α* = .45 and *α* = .55 for neutral stories (the latter was low due to floor effects for neutral stories).

#### 2.3.3 State trait anxiety inventory (STAI)

The state version of the STAI (Spielberger, 1983), a widely used standardized measure of state anxiety was given at baseline and after each block of picture stories. The difference between baseline and post trauma-block scores indexed the anxiety response to the trauma stories.

#### 2.3.4 Intrusion interview

Intrusive memories of the traumatic picture stories were assessed by telephone interview 2 weeks and 3 months after the experiment. Participants were asked whether any unwanted images from the picture stories had popped into their mind during the last week and if so, to describe their content (e.g., a spontaneously occurring mental image of the child that featured in one story). Overall frequency of intrusive memories was rated on a scale from 0 to 7 (*never, once, twice, three to four times, about every other day, nearly every day, more often than once a day*). Distress for each intrusive memory was assessed on a scale from 0 *not at all distressing* to 10 *very much distressing*.

#### 2.3.5 National adult reading test (NART)

After the picture stories, participants completed the NART (Nelson & Willison, 1982), a measure of verbal intelligence that requires participants to read out aloud a list of 50 irregularly spelt words. The number of words pronounced correctly comprises the final score. The NART correlates highly with other measures of intelligence and allows the prediction of full-scale IQ scores (Blair & Spreen, 1989).

### 2.4 Procedure

The study was approved by the local ethics committee. On arrival at the laboratory participants gave written consent. They then filled in the STAI and provided information about demographics, and past experiences with traumatic events, similar experiments or distressing visual material. Afterwards the first block of the picture stories (trauma or neutral) was shown, followed by a 10-min break when participants completed the DDPS, SDQ and STAI. The investigator conversed with the participant about unrelated matters until the 10 min were over. After presentation of the second block of picture stories (trauma or neutral), participants completed the DDPS, SDQ, and STAI again, followed by the NART and two other filler tasks. The *perceptual priming task*, *manipulation check interview* and *object recognition task* followed. Afterwards the experimenter explained further steps of the study and reimbursed the participant. The investigator confirmed that the participant felt well and encouraged them to get in contact in case they felt distressed about the experiment. However, none of the participants took up this offer. The *Intrusion Interview* was conducted on the telephone 2 weeks and 3 months later.

### 2.5 Data analysis

Statistical analyses were performed with SPSS version 19. We report two-tailed levels of significance; values of *p* < .05 were considered significant. The main analyses were repeated measures analyses of variance (ANOVA), with story context (trauma vs. neutral) as the within-subject factor. Following Rosenthal (1995), effect sizes were computed as ${\eta_{p}}^{2}$, which reflects the proportion of total variability attributable to a factor when all other factors are taken into account. Effects of $.01<{\eta_{p}}^{2}<.0588$ are considered small, $.0588\geq {\eta_{p}}^{2}\leq .1379$ medium and ${\eta_{p}}^{2}\geq .1379$ large (Cohen, 1988, p. 283).

## 3 Results

### 3.1 Validity of the picture stories

As expected and in line with pilot study 3, participants perceived trauma stories as significantly more unpleasant (*M* = −6.4, *SD* = 2.7 vs. *M* = 5.4, *SD* = 3.2; *F*(1, 50) = 408.0, *p* < .001, ${\eta_{p}}^{2}=.89$) and activating (*M* = 3.6, *SD* = 3.7 vs. *M* = −4.2, *SD* = 3.7; *F*(1, 50) = 175.8, *p* < .001, ${\eta_{p}}^{2}=.78$) than neutral stories. In addition, participants reported significantly greater state anxiety during traumatic picture stories, *M* = 35.9, *SD* = 9.2, compared to neutral stories, *M* = 29.3, *SD* = 7.8; *F*(1, 50) = 17.1, *p* < .001, ${\eta_{p}}^{2}=.31$, and baseline, *M* = 30.4, *SD* = 6.4; *F*(1, 50) = 22.56, *p* < .001, whereas state anxiety during neutral stories did not differ from baseline, *F*(1, 46) = 1.53, *p* = .22.

Participants engaged significantly more in data-driven processing (*M* = 0.9, *SD* = 0.6 vs. *M* = 0.6, *SD* = 0.4; *F*(1,50) = 9.4, *p* = .004, ${\eta_{p}}^{2}=.16$) and state dissociation (*M* = 0.3, *SD* = 0.4 vs. *M* = 0.1, *SD* = 0.2; *F*(1,50) = 10.4, *p* = .002, ${\eta_{p}}^{2}=.18$) during trauma stories than during neutral stories.

### 3.2 Perceptual priming task

#### 3.2.1 General priming effect

To check for a general priming effect, a repeated measures ANOVA compared identification rates for all objects that participants had previously seen interspersed into the picture stories with those for new objects. Primed objects were more readily identified, *M* = .60, *SD* = .14, than unprimed objects, *M* = .40, *SD* = .15, *F*(1, 50) = 101.0, *p* < .001, ${\eta_{p}}^{2}=.70$.

#### 3.2.2 Effects of trauma context on priming (Hypothesis 1)

Table 2 shows the effects of story context on object identification in the blurred picture task. In line with Hypothesis 1, objects previously seen in the context of trauma stories were identified with greater probability than objects previously seen in the context of neutral stories.

##### 3.2.2.1 Further analyses

Twenty-six participants (51%) reported having tried to *actively recall* the objects during the priming task; identification rates *active recall*: *M*_trauma_ = .66, *SD*_trauma_ = .15; *M*_neutral_ = .65, *SD*_neutral_ = .13; *no active recall*: *M*_trauma_ = .60, *SD*_trauma_ = .18; *M*_neutral_ = .52, *SD*_neutral_ = .14. A 2 × 2 ANOVA with story context (trauma vs. neutral) as the within factor and active recall (yes vs. no) as the between factor, showed a main effect for active recall, *F*(1, 49) = 6.41, *p* = .015, ${\eta_{p}}^{2}=.12$, indicating that participants who used the active recall strategy had significantly higher identification rates than those who did not. Further, there was a trend for an interaction Story Context × Active Recall, *F*(1, 49) = 2.61, *p* = .113, ${\eta_{p}}^{2}=.05$. Bonferroni-controlled post-hoc analyses revealed that participants who did not actively recall the objects identified objects from trauma stories significantly more often than objects from neutral stories, *F*(1, 49) = 6.9, *p* = .011, ${\eta_{p}}^{2}=.12$, whereas no significant difference was found in the active-recall group, *F*(1, 49) = .15, *p* = .703, ${\eta_{p}}^{2}=.00$. Therefore, active recall was controlled for in the following analyses where appropriate.

To test whether order of story block influenced identification rates, a mixed 2 × 2 ANOVA with order (trauma stories vs. neutral stories presented first) as the between factor and story context (trauma vs. neutral) as the within factor and was computed. There was no main effect for order, *F*(1, 49) = .005, *p* = .946, ${\eta_{p}}^{2}=.00$; but the main effect of story context, *F*(1, 49) = 5.46, *p* = .047, ${\eta_{p}}^{2}=.078$, was qualified by a significant Order × Story Context interaction, *F*(1, 49) = 5.46, *p* = .024, ${\eta_{p}}^{2}=.10$. Post hoc analyses revealed that the EPP effect was only significant for participants who saw the trauma stories first, *F*(1,49) = 10.16, *p* = .001, ${\eta_{p}}^{2}=.17$; and could not be demonstrated for those who saw the neutral stories first, *F*(1,49) = .042, *p* = . 838, ${\eta_{p}}^{2}=.00$. Therefore, order of story block was controlled in the following analyses where appropriate.

### 3.3 Recognition task

Table 2 shows the results of the object recognition task. As expected, there were no main effects or interactions of story context for sensitivity of discrimination (*d*′) or response bias (*c*). Equally there were no differences in hits (both 70%) or false alarm rates (25–27%) for the two story contexts. Additional analyses found no main effects or interactions for order of story block.

### 3.4 Predictors of intrusive memories

At 2 weeks 10 participants (19.6% of 51) reported intrusive memories of images from the picture stories in the preceding week with mean distress ratings of *M* = 5.7, *SD* = 2.4, range = 2–9; mean intrusion frequency *M* = .43, *SD* = .99, range = 0–4. At 3 months, 5 participants (12.5% of 40) reported intrusions with mean distress ratings of *M* = 5.5, *SD* = 1.9, range = 4–8: mean intrusion frequency *M* = .5, *SD* = 1.45, range = 0–7. In addition, a few participants reported intrusions of the interspersed neutral objects, but these were not included in the correlational analyses as these may have been influenced by the two memory tests (Krans, Näring, Holmes, & Becker, 2009). In accordance with Hypothesis 2, EPP predicted intrusive memories in the second week after the experiment, *r* = .29, *p* = .04. This relationship remained significant when controlling for order of story block, *r* = .29, *p* = .04 (trauma stories first, *r* = .32; neutral stories first, *r* = .30), and active recall, *r* = .29, *p* = .04. However, EPP did not predict intrusions at 3 months, *r* = .02, *p* = .91, *n* = 40.

As expected, recognition sensitivity (*d*′) for objects from trauma stories did not correlate with intrusive memories reported at 2 weeks, *r* = −.05, *p* = .710. There was a trend for a negative relationship with intrusions at 3 months, *r* = −.28, *p* = .076.

In line with Hypothesis 3, both data-driven processing, *r* = .33, *p* = .018, and state dissociation during trauma stories, *r* = .53, *p* < .001, predicted the frequency of intrusive memories in the second week after the experiment. Data-driven processing and dissociation no longer significantly predicted intrusive memories at 3 months (*r*'*s* < .21, *p*'*s* > .20). Increases in state anxiety with the trauma stories also predicted intrusive memories at 2 weeks, *r* = .36, *p* = .012, but not at 3 months, *r* = −.07, *p* = .68. There were no significant correlations with intelligence.

A multiple regression analysis tested the relative contribution of EPP, recognition sensitivity, data-driven processing and dissociation, pleasantness and arousal ratings, anxiety response, and verbal intelligence (NART) to the prediction of intrusive memories at 2 weeks. The predictors were entered simultaneously and explained 40.6% of the variance, *R* = .637, *F*(8, 45) = 3.16, *p* = .008. EPP, *β* = .30, *p* = .041, explained unique variance; there was a trend for intelligence, *β* = .24, *p* = .098, but no unique effects of anxiety, *β* = .24, *p* = .124, arousal *β* = −.22, *p* = .157, pleasantness, *β* = −.113, *p* = .457, and recognition sensitivity, *β* = .01, *p* = .942. Data-driven processing, *β* = .247, *p* = .181, and dissociation, *β* = .249, *p* = .177, which are conceptually related measures, correlated substantially, *r* = .59, and did not explain unique variance when they were both entered together into the multiple regression. When they were entered individually, they each explained unique variance over and above the other predictors, data-driven processing, *β* = .374, *p* = .023, and dissociation, *β* = .374, *p* = .023.

### 3.5 Predictors of enhanced perceptual priming

In contrast to Hypothesis 4, EPP did not correlate with self-reported data-driven processing, *r* = −.03, *p* = .836, nor with state dissociation, *r* = .19, *p* = .182. Controlling for order of story block and active recall did not affect these relationships. Other reactions during the trauma stories, including increases in state anxiety, perceived arousal and pleasantness did not correlate with EPP either (see Table 3). The only variable that predicted EPP was lower verbal intelligence as measured by the NART, *r* = −.30, *p* = .034. Lower intelligence also correlated with data-driven processing, *r* = −.31, *p* = .027.

## 4 Discussion

This analogue study supports the role of both perceptual processing during exposure to traumatic material and enhanced perceptual priming for stimuli perceived in a traumatic context in subsequent intrusive memories.

### 4.1 Enhanced perceptual priming in traumatic contexts

Consistent with Hypothesis 1, neutral objects were more strongly primed if participants saw them in the context of traumatic picture stories than if they saw the same objects in the context of neutral stories. In accordance with Hypothesis 2, EPP for objects seen in traumatic contexts predicted intrusive memories 2 weeks after the experiment. Although EPP effect sizes were only in the moderate range $\left({\eta_{p}}^{2}=.08\right)$ and influenced by order effects, the findings replicate and extend earlier findings demonstrating the effects of traumatic contexts on perceptual priming and its relationship with subsequent intrusive memories (Ehlers et al., in press; Ehlers et al., 2006; Michael & Ehlers, 2007). The novel paradigm used in the present study allowed us to demonstrate the EPP more unambiguously than in the previous studies as the objects for which priming was measured were no longer embedded within the stories and could therefore be counterbalanced across story contexts. The results underline clinical observations that trauma triggers and intrusive memories may include stimuli that do not have a meaningful relationship with the trauma and are merely linked to the trauma by temporal association (e.g., Ehlers et al., 2002).

There has been a debate in the literature on the influence of explicit memory processes on the performance of implicit memory tasks, such as the perceptual priming test employed in this study (Jacoby, Toth, & Yonelinas, 1993). The current study was not designed to address this issue, but the pattern of findings suggests that the EPP effect and its relationship with subsequent intrusions was not mediated by explicit memory. Story context did not affect recognition performance in the object recognition task, and participants did not exhibit a differential response bias for the stories. This makes it unlikely that the EPP effect stems from explicit memory processes. Secondly, participants who used active recall strategies during the blurred picture task showed a reduced EPP effect (i.e., a smaller difference between objects from trauma and neutral stories), suggesting that active recall masked the EPP effect. The priming test results for participants who used active recall resemble those for the recognition memory test. If the EPP effect was due to explicit memory processes, one would have expected enhancement of EPP with active recall. Furthermore, recognition sensitivity was not related to intrusions at 2 weeks and even tended to be negatively related to intrusions at 3 months. Since strong explicit memory indicates better elaboration (Baddeley, 1997), this finding is in accordance with Ehlers and Clark's (2000) proposal that elaboration enhances higher-order meaning-based retrieval strategies and at the same time inhibits direct triggering of intrusive memories (see also the effects of experimentally-induced elaboration, Ehlers et al., in press; Michael & Ehlers, 2007).

EPP did not predict intrusive memories at 3 months. The choice of this time point may not have been ideal as the trauma stories used in this study were only a weak stressor and may not be distressing enough to induce long-lasting intrusions. Very few participants reported intrusions at 3 months. Thus, this result may indicate a floor effect. Furthermore, the sample was reduced by 3 months, which resulted in low power. Alternatively, the results may also indicate post-experiment conceptual processing of the stimulus material that may have led to a decrease in the EPP effect over time (Ehlers et al., in press; Michael & Ehlers, 2007). It is also conceivable that the recognition test may have contributed to an overall reduction in intrusive memories (Krans et al., 2009).

So far, it remains unclear what factors underlie the observed EPP effect for trauma-related stimuli. The results are in line with other studies showing that task relevance can influence perceptual priming (Holbrook, Bost, & Cave, 2003), suggesting that priming may not just be an automatic consequence of perception but may reflect a more adaptive and flexible mechanism for the modification of perceptual processing. Therefore, it can be speculated that EPP for trauma-related cues may be an adaptive response that helps the individual to identify similar cues and thus avoid potentially dangerous situations in the future (Ehlers et al., 2002). This proposed function of EPP is similar to that of fear conditioning which is considered to be essential for survival, as it helps identify reliable predictors of significant events and the triggering of preparatory responses (Davey, 1987; Dawson & Schell, 1987). Although EPP and fear conditioning serve adaptive purposes they may come at cost when their inappropriate activation increases the probability that harmless cues in the environment trigger reexperiencing symptoms in the aftermath of a trauma (Ehlers et al., 2006).

### 4.2 Perceptual processing during the traumatic picture stories

In line with Hypothesis 3, data-driven processing and dissociation predicted intrusive memories at 2 weeks. Effect sizes were small, but in line with previous studies (e.g., Halligan et al., 2003; Holmes et al., 2004; Murray et al., 2002). In interpreting the effect sizes, one has to bear in mind that trauma stories were only moderately aversive and means for data-driven processing and dissociation were low. Both data-driven processing and dissociation (when considered individually) contributed unique variance to the prediction of intrusive memories that was not explained by the other predictors such as anxiety or arousal. This suggests that the way people process traumatic material affects the probability of subsequent reexperiencing symptoms beyond what is explained by high levels of emotion. Data-driven processing and dissociation have in common that they lead to preferential encoding of perceptual information, which may facilitate the subsequent triggering of intrusive memories by matching perceptual cues (Ehlers & Clark, 2000). Correlations were no longer significant at 3 months, probably due to floor effects and the restricted range of perceptual processing.

In contrast to Hypothesis 4, data-driven processing and dissociation during the picture stories did not correlate with EPP. Several possible explanations are conceivable. First, self-reports may only be imprecise assessments of perceptual processes as they rely on introspection and individuals may differ in their ability to consciously access such complex cognitive processes. Second, objects were shown *before* picture-onsets to allow for counterbalancing within and across picture stories. However, this methodological advantage might have come at a cost to the precision of assessing processing of the objects for which priming was later measured. It is conceivable that the self-reports reflected perceptual processing during the trauma pictures, but not during the presentation of the objects. Third, theoretical assumptions about priming as the pathway by which perceptual processing leads to intrusions might not be correct. An alternative pathway might be a poorly elaborated trauma memory (Ehlers & Clark, 2000). More research is needed to examine the causal relationship between cognitive processes and priming.

Interestingly, both data-driven processing and EPP were predicted by low verbal intelligence. Low verbal intelligence is one of the risk factors for PTSD (McNally & Shin, 1995). Ehlers and Clark (2000) speculated that low intelligence may increase the risk for PTSD by reduced conceptual and enhanced perceptual processing during trauma. The current findings are in line with this hypothesis.

### 4.3 Limitations

The present study had limitations. Firstly, the study employed an analogue design and the extent to which results generalize to real-life trauma and PTSD reexperiencing remains unclear. Nevertheless, analogue designs are generally seen as a suitable way to induce and investigate mechanisms underlying intrusive reexperiencing (Ehring, Kleim, & Ehlers, 2011; Holmes & Bourne, 2008). Second, the overall number of intrusive memories was low. This may reflect the fact that participants found the stories only moderately distressing. Furthermore, we measured intrusions during the past week retrospectively over the phone, thus possibly underestimating their frequency. A phone interview was chosen over a diary to ensure participants understood the distinction between intrusive memories and intentionally retrieved memories and that the intrusions were about images from the stories rather than other aspects of the experiment. Thirdly, error variance was increased by an effect of order of story block. Participants who first watched trauma stories exhibited a significant EPP effect, whereas participants who first watched the neutral stories did not show this effect. This problem might have arisen because the current study did not familiarize participants with the paradigm by using a practice story. Participants may therefore have processed the first picture story and its interspersed neutral objects differently from the following stories. Although participants were instructed to focus on the stories and “not to take special notice” of the interspersed objects, it is conceivable that they paid more attention to these objects in the first block of stories than in the second block. Strength of priming has been found to be greatest for those items that receive the most attention (Stankiewicz, Hummel, & Cooper, 1998), which may explain the significant interaction between ‘order of story block’ and story context. Future studies should address this problem by using a practice story. Fourth, the assessment of perceptual processing was limited to self-reports, and it would be of interest to include objective measures of attention to perceptual and conceptual aspects of the stimulus material.

Finally, although great care was taken in matching the picture story conditions in many respects, the comparison of neutral and trauma stories did not allow us to investigate the specificity of EPP to trauma stories. Future studies should investigate whether EPP also occurs in equally arousing but non-aversive contexts, or equally negative but non-arousing contexts. However, the specificity of the EPP effect is *not* of theoretical relevance to the model of intrusive trauma memories investigated here (Ehlers & Clark, 2000), as this model does *not* claim specificity. It is conceivable that EPP may be a mechanisms involved in triggering unintentional memories of emotional events in general.

### 4.4 Conclusions and future directions

The results support the role of perceptual processing and perceptual priming in reexperiencing symptoms. They are in line with information processing models of PTSD that emphasize strong encoding of perceptual information and relatively weak encoding of contextual information during trauma as risk factors for subsequent reexperiencing symptoms (Brewin et al., 2010; Ehlers & Clark, 2000). Further studies are needed to clarify the role of perceptual priming and perceptual processing in the development of intrusive memories after trauma. Avenues to build on the current findings may be to employ more sensitive measures of visual priming such as reaction times, use of more distressing stimuli or clinical populations, and manipulations of cognitive processing to generate more direct experimental evidence on hypothesized relationships between processing styles, priming and intrusions (see Holmes et al., 2004; Kindt, Van den Hout, Arntz, & Drost, 2008; Stuart, Holmes, & Brewin, 2006). Finally, clinical observations show that intrusions often reflect stimuli that signalled the onset of the trauma or of its worst moments (Ehlers et al., 2002; Hackmann et al., 2004). Interestingly, some participants reported that they had intrusive memories of the interspersed objects rather than the picture stories themselves. Therefore, it would be of interest to systematically measure intrusions from the unrelated interspersed neutral objects in future studies.

## Figures and Tables

**Table 1:** Structure of picture stories: example of one trauma story and parallel neutral story.

Part of story	Picture	Trauma story	Neutral story
Neutral introduction (identical for trauma and neutral story)	1	“George Miller” lies in bed with his wife, reading a book	“George Miller” lies in bed with his wife, reading a book
	2	George works at his desk preparing a presentation	George works at his desk preparing a presentation
	3	George stays up late, working	George stays up late, working
Main part, content either traumatic or neutral	4	Burglar breaks into the house	George has breakfast
	5	Masked man appears in front of George, holding a knife	George leaves the house
	6	George's throat is cut, bleeding badly	Journey to work, lots of traffic
	7	Ambulance rushes George to the hospital, but George dies	At office, George talks with colleagues
	8	George's funeral	George gives his presentation

**Table 2:** Results of priming and recognition tests.

Memory task		Picture stories		Statistic		
		Trauma	Neutral	*F*(1,50)	*p*	${\eta_{p}}^{2}$
Priming task	Identification score	.63 (.16)	.58 (.15)	4.40	.042	.080
Recognition task	Sensitivity (*d*′)	1.40 (.96)	1.50 (1.01)	.74	.395	.014
	Response bias (*c)*	.03 (.51)	.05 (.47)	.11	.740	.002

**Table 3:** Correlations between variables in multiple regression analysis.

	2	3	4	5	6	7	8	9
1. Intrusions at 2 weeks	.29*	−.05	.33*	.53***	.36*	.05	.28	.03
2. Enhanced Priming		.14	−.03	.19	.04	−.05	.06	−.30*
3. Recognition sensitivity			−.20	−.13	−.15	−.05	.12	.02
4. Data-driven processing				.59***	.30*	.29*	−.40**	−.31*
5. Dissociation					.44**	.24	−.42**	−.20
6. Anxiety						.45**	−.31*	−.06
7. Arousal							−.38**	−.14
8. Pleasantness								.16
9. Verbal Intelligence								

*p < .05, **p < .01, ***p < .001.
